# Effect of marker placement on Oxford Foot Model hindfoot segment axes

**DOI:** 10.1186/1757-1146-7-S1-A62

**Published:** 2014-04-08

**Authors:** Adward MH Paik, Julie Stebbins, Alpesh Kothari, Amy B Zavatsky

**Affiliations:** 1 Department of Engineering Science, University of Oxford, Oxford, OX1 3PJ, UK; 2 Oxford Gait Laboratory, Nuffield Orthopaedic Centre, Oxford, OX3 7LD, UK; 3 Nuffield Department of Orthopaedics Rheumatology and Musculoskeletal Sciences, Nuffield Orthopaedic Centre, Windmill Road, Oxford, OX3 7LD, UK

## Background

Measurement accuracy of joint kinematics is influenced by how closely the trajectories of surface markers represent the motion of underlying bones [1]. For the Oxford Foot Model (OFM) hindfoot segment, the heel (HEE), lateral calcaneus (LCA) and sustentaculum tali (STL) markers are used to track the movement of the calcaneus [2]. This paper investigates the how changes in marker placement affect the orientation of the OFM hindfoot segment axes.

## Methods

Twenty adult females participated in the study (40 feet in total). Radiopaque monitoring electrodes (Type 2223, 3M Healthcare, Neuss, Germany) were placed on the feet at the locations specified by the OFM. CT images (GE 64-slice Lightspeed VCT scanner) were acquired as the subjects lay supine. The 3-Dimensional (3D) coordinates of the electrodes and of the points corresponding to the ideal marker locations were extracted from the images, using Mimics (Materialise NV, Leuven, Belgium). The marker based OFM A-P axis (AP) which extends from HEE to the mid-point (MID) of LCA and STL was calculated. A corrected A-P axis based on the ideal HEE marker location (APH) was also calculated. MID was then adjusted by correcting either LCA or STL to make them equidistant from HEE, as specified by the OFM marker placement protocol. From that, a corrected A-P axis based on the modified MID (APM) was computed. Finally, a fully corrected A-P axis (APC) based on the revised HEE and MID positions were obtained. The transverse plane projections of all the axes (AP, APH, APM, APC) were compared.

## Results

The results (Table 1) suggest that correcting the position of either the LCA or the STL marker induced less than 1° of change in the anterior-posterior (A-P) axis for most feet. Whereas, when the HEE marker position was aligned with the correct anatomical location, the orientation of the A-P axis was affected more as both the mean and interquartile (IQR) values infer. There was large variation in its orientation relative to the original A-P axis with a slight medial bias. From regression analysis it was found that, 1 mm of lateral shift in HEE placement was enough to cause approximately 4° of deviation in the A-P axis orientation.

**Table 1:** HEE and MID placement error and angular differences between the AP, APH, APM, and APC axes.

	APM – AP(°)	APH – AP (°)	APC – AP (°)	MID Error (mm)	HEE Error (mm)
**Mean**	-0.4	-2.1	-2.5	1.8	1.9

**Median**	0.0	0.0	-0.2	1.7	1.7

**IQR**	1.3	14.1	15.4	8.7	6.3

## Conclusion

The anteroposterior orientation of the A-P axis is more sensitive to the location of the HEE marker than to the locations of the LCA and STL markers. Therefore, it is essential to ensure that the HEE marker is placed accurately.
